# Tissue-specific expression and functional role of keratin 1 in sheep horn development

**DOI:** 10.3389/fvets.2025.1703700

**Published:** 2025-12-15

**Authors:** Qiao Mei, Hao Li, Guoqing Zhang, Zhu Meng, Shiwen Zhang, Fei Liu, Zhangyuan Pan, Jie Yang

**Affiliations:** 1 College of Life Science and Technology, Xinjiang University, Ürümqi, China; 2 State Key Laboratory of Animal Biotech Breeding, Institute of Animal Science, Chinese Academy of Agricultural Sciences, Beijing, China; 3 National Nanfan Research Institute (Sanya), Chinese Academy of Agricultural Sciences, Sanya, China

**Keywords:** KRT1, sheep horn, RNA-seq, ASE, WGS, SNPs

## Abstract

**Introduction:**

Sheep horn development has significant implications for animal welfare and farm management, yet its molecular mechanisms remain incompletely understood. Keratin 1 (KRT1), a key structural protein in epidermal keratinization, has been implicated in horn formation. This study systematically investigates the expression patterns, genetic variations, and functional role of KRT1 in sheep horn development.

**Methods:**

We integrated RNA sequencing (RNA-seq) and whole-genome sequencing (WGS) data from Tibetan sheep and public databases. Multi-tissue expression profiling of KRT1 was performed across sheep, cattle, pigs, and humans. Phylogenetic and protein structural analyses identified conserved amino acid sites. Allele-specific expression (ASE) loci and functional SNPs were screened using population genetics approaches. Association analysis linked genotypes with horn length in Small Tail Han sheep.

**Results:**

KRT1 expression was significantly higher in scurred (small-horned) sheep compared to SHE (large-horned) sheep (*p* = 0.024), and exhibited tissue-specific enrichment in horn, skin, and periosteum. Cross-species analysis confirmed high KRT1 expression in horn and skin tissues. We identified 11 horned-artiodactyl-specific amino acid sites, including K312, which forms a hydrogen bond with E262 of KRT10; mutations at this site disrupted the interaction. Four ASE loci showed strong bias toward reference alleles in horned phenotypes. Thirty-two functional SNPs were prioritized, and nine haplotype blocks contained 13 highly differentiated SNPs (Fst > 0.05). Four SNPs were significantly associated with horn length, with wild-type homozygotes exhibiting longer horns (*p* < 0.05).

**Discussion:**

Our findings demonstrate that KRT1 plays a critical role in sheep horn development through its expression regulation, protein interaction stability, and genetic variation. The conserved K312 residue and associated SNPs may serve as potential molecular markers for horn phenotype selection. These results provide new insights into the keratin-based mechanisms underlying horn morphogenesis and offer a foundation for molecular breeding strategies aimed at horn size and type modulation in sheep.

## 1 Introduction

Sheep (*Ovis aries*) are globally important livestock, providing humans with high-quality mutton, nutritious milk, and wool, a key raw material for the textile industry (1). In intensive farming systems, maximizing production efficiency and ensuring animal welfare are core objectives. However, sheep horns—an anatomical structure composed of a bony core and an outer keratin sheath (2)—pose significant challenges in densely housed environments. In natural settings, horns play important roles in defense against predators, intraspecific competition, and sexual selection (3, 4). Under confined conditions, however, fighting among horned individuals frequently causes severe injuries, such as body surface bruising, udder damage (reducing milk yield), and deterioration of carcass quality (affecting meat quality) (5). These traumas not only directly impact individual growth, development, and reproductive performance but also increase the risk of injury to handlers during routine management operations (6). To mitigate these issues, early physical disbudding is commonly practiced in farming. However, this procedure is typically performed on lambs within 1–2 weeks after birth and often lacks effective analgesia due to technical limitations, safety concerns, and regulatory requirements, causing significant pain to the animals and constituting a serious animal welfare problem (7). Furthermore, routine disbudding has been banned in some regions, only permitted under specific conditions (8). Therefore, deciphering the molecular mechanisms of horn formation and development is crucial for enhancing animal welfare and ensuring sustainable production benefits in the livestock industry.

The *KRT1* gene primarily encodes Keratin 1, a key structural protein in epidermal keratinization. Existing research indicates that KRT1 is closely associated with the formation of the keratin sheath and plays an important role in its development (9). Sheep horns, as a specialized organ, consist of an internal bony core and an external keratin sheath (2). Studies on sheep horn type differentiation have identified several key genes, including *RXFP2, FOXL2, ACAN*, and *TNN*, all of which have been confirmed to significantly influence horn phenotypes (10). Among these, the relaxin family peptide receptor 2 (*RXFP2*) gene, located on chromosome 10 and enriched in the neuroactive ligand-receptor interaction pathway, is considered a key gene controlling horn formation and is significantly associated with horn size and shape (11, 12). Notably, the *KRT1* gene has also been identified as a candidate gene for horn development, potentially influencing sheep horn size (10). Homozygous nonsense mutations in *KRT1* [e.g., *c.457C*>*T* (*p. Gln153) and c.33C*>*G (p. Tyr11*)] can lead to nonsense-mediated mRNA decay and KRT1 protein deficiency, resulting in epidermolytic palmoplantar keratoderma (EPPK) with knuckle pads (13); additionally, *KRT1* mutations have been confirmed to cause epidermolytic hyperkeratosis, manifesting as ichthyosis-like hyperkeratosis (14). These findings consistently demonstrate the central role of *KRT1* in epidermal keratinization. Given that horns are skin derivatives and the development of their keratin sheath is primarily regulated by skin-related pathways (15), we hypothesize that the *KRT1* gene may regulate the generation, development, and final morphology of sheep horns by influencing skin keratinization pathways.

Based on this background, this study aims to systematically dissect the core regulatory role and molecular mechanism of the *KRT1* gene in sheep horn morphological differentiation. To achieve this, we integrated RNA-seq transcriptome sequencing and whole-genome sequencing (WGS) data to conduct multi-dimensional analyses: (1) Investigate the differential expression patterns of the *KRT1* gene in horn tissue and other key tissues (e.g., periosteum, skin, internal organs); (2) Systematically identify functional genomic variations affecting horn development [including allele-specific expression (ASE) loci, functional SNP loci, and linkage disequilibrium haplotype blocks]; (3) Conduct in-depth analysis of key amino acid loci and their mediated protein interaction networks (e.g., KRT1-KRT10 interaction). Through these integrated analyses, this study aims to elucidate the molecular pathway by which the *KRT1* gene influences horn morphological development through regulating the keratin interaction network. This will provide crucial new evidence for a deeper understanding of the molecular basis of sheep horn development and lay the theoretical foundation for molecular marker-assisted breeding based on functional loci within the *KRT1* gene.

## 2 Materials and methods

### 2.1 Ethical statement

The animal study protocol was approved by the Animal Ethics Committee of the Institute of Animal Sciences, Chinese Academy of Agricultural Sciences (IAS-CAAS) under approval number IASCAAS-AE-03 (December 12, 2016).

### 2.2 Animals and sample collection

The RNA-seq dataset used in this experiment was previously collected by our research group (PRJNA1003277) (16). Soft horn tissue samples were collected from eight female Tibetan sheep (with an average age of 4.5 years) in the Tibet region of China. Samples were divided into two groups: the scurred group (small horns, *n* = 4, horn length 0–12 cm) and the SHE group (large horns, *n* = 4, horn length >12 cm; Supplementary Table S1). The collected tissues were placed in cryotubes and flash-frozen in liquid nitrogen.

### 2.3 RNA-seq data analysis

To explore the expression of the *KRT1* gene across tissues, 2,915 publicly available high-quality RNA-seq datasets from sheep were collected, primarily sourced from the National Center for Biotechnology Information (NCBI) database (https://www.ncbi.nlm.nih.gov/, accessed on July 23, 2023) and the European Bioinformatics Institute (EBI) database (https://www.ebi.ac.uk/, accessed on July 23, 2023). These public datasets, along with the RNA-seq data from the 8 Tibetan sheep collected previously, were processed as follows. Adapters and low-quality data were removed using Trim Galore (v.0.6.10). Clean reads were mapped to the sheep reference genome ARS-UI_Ramb_v2.0 using STAR (v.2.7.10b) with parameters –chimSegmentMin 10 and –outFilterMismatchNmax 3. High-quality RNA-seq clean data meeting the criteria of unique mapping rate >85% and number of clean reads >20,000,000 were obtained for subsequent analysis (17) (Supplementary Table S2). Transcripts Per Million (TPM) and Fragments Per Kilobase of exon per Million mapped fragments (FPKM) values were calculated using StringTie (v.2.1.5) (18). Raw gene counts were subsequently extracted using featureCounts (v.2.0.1).

### 2.4 KRT1 gene expression in SHE and scurred groups

To explore differences in *KRT1* gene expression between the SHE and scurred groups, violin plots were generated using the ggplot2 (v.3.4.2) package in R (v.4.3.1), and significance was assessed using a *T*-test. To further investigate differences in exon expression of the *KRT1* gene between the SHE and scurred groups, we used Subread_to_DEXSeq (https://github.com/vivekbhr/Subread_to_DEXSeq) of dexseq_prepare_annotation2.py script to format the annotation (GTF) file of the genome, then used load_SubreadOutput.R in Rstudio reads the formatted GTF file and the counts' matrix output by featureCounts, constructs the DEXSeqDataSetFromFeatureCounts (dds) object, and performs exon difference analysis (19). To further visualize the differential expression of the *KRT1* gene across the SHE group, scurred group, and different tissues, the *KRT1* gene track was constructed and visualized on the UCSC Genome Browser (https://genome.ucsc.edu/s/yoser4/15horn_vs_145other).

### 2.5 KRT1 gene expression profiling

To explore *KRT1* gene expression across species, publicly available RNA-seq data from four species (sheep, pig, cattle, and human) were downloaded. RNA-seq data from 2,651 pigs were obtained from the Pig GTEx project, with TPM gene expression values sourced from http://piggtex.farmgtex.org/ (accessed 10 September 2023) (20). RNA-seq data from 4,359 cattle were obtained from the Cattle GTEx project, with TPM gene expression values sourced from https://cgtex.roslin.ed.ac.uk/ (accessed 1 October 2023) (21). TPM gene expression values for RNA-seq data from 9,810 human samples were sourced from https://gtexportal.org/home/datasets (accessed 5 August 2023) (22, 23). Sheep, pig, cattle, and human RNA-seq data were pooled to align tissue samples, which were subsequently categorized into 17 distinct tissue types. The average TPM value of the *KRT1* gene for each species was calculated. Bubble plots illustrating the tissue-specific expression of the *KRT1* gene across the four species were generated using the pheatmap (v.1.0.12) package in R (v.4.3.1). Additionally, to investigate sex-specific differences in tissue expression of the sheep *KRT1* gene, significant differential expression between rams and ewes across 12 tissues was calculated using a *T*-test. To study differences in *KRT1* gene expression between scurred and SHE sheep breeds, data for the seven most representative sheep breeds from the aforementioned dataset were extracted and divided into horned and hornless groups for expression analysis. The groups were defined as follows: horned Group: Minxian Black Fur Sheep, Tibetan Sheep, Tan Sheep, Chinese Merino Sheep; hornless Group: Bashibai Sheep, Texel Sheep, Hu Sheep, using multiple comparisons test.

### 2.6 KRT1 protein sequence analysis and 3D structure prediction

To investigate differences in the KRT1 protein among different species, protein sequences of KRT1 homologs from 18 distinct species were retrieved from the NCBI Homologene database (Supplementary Table S3). Using MEGA11 software (24), multiple sequence alignment was performed using the ClustalW method (default parameters) (25). Based on the alignment results, a circular phylogenetic tree of the KRT1 protein was constructed using the Neighbor-Joining (NJ) method (26). Differences in the KRT1 protein sequences across species were analyzed, with a particular focus on differences between horned artiodactyl species and other species, to identify specific amino acid loci. The three-dimensional structure of the KRT1 protein was predicted using AlphaFold3 (default parameters) via NCBI, and the spatial locations of the specific amino acid loci were annotated (27). To identify spatial binding loci of the KRT1 protein, a Protein-Protein Interaction (PPI) network was constructed using the STRING database (28) to predict the interaction network of the KRT1 protein in sheep. The three-dimensional structure of the interaction between the KRT1 and KRT10 proteins was predicted using AlphaFold3 (default parameters). PyMOL was used for visualization analysis (29). Combining the specific amino acid loci identified above, the specific amino acid loci mediating the interaction between KRT1 and KRT10 proteins were identified. The effect of mutations at these specific binding loci amino acids on the hydrogen bonding interaction between the two proteins was observed. The resulting mutant protein complex structures were predicted using AlphaFold3 software and visualized using Pymol.

### 2.7 Whole-genome sequencing (WGS) analysis

Whole-genome sequencing (WGS) data comprised publicly available data and data collected in the laboratory. Publicly available data for 200 sheep were downloaded from NCBI, including projects PRJNA304478, PRJNA325682, PRJNA47952, PRJNA624020, PRJNA675420, PRJNA822017, PRJNA30931, PRJNA480684, PRJNA509694, PRJNA779188, and PRJNA783661 (30–41). Samples were classified as horned or polled. The FST values—a measure of genetic differentiation between populations—were calculated for samples from the public data using vcftools (v.0.1.16) with default parameters. An FST value >0.05 was set as the differentiation threshold. SNPs were annotated using SnpEff and categorized based on the annotation results (e.g., exon, intron, upstream, downstream) to filter for more specific SNP loci in these sheep. Linkage disequilibrium (LD) analysis of SNP loci within the *KRT1* gene region was performed, and LD heatmaps were generated using LDBlockShow (v.1.40) software (42).

To validate the WGS findings, data from 35 Small Tail Han sheep were collected (horn length trait was defined as the average of left and right horn lengths). Raw data were trimmed and filtered for low-quality sequences and adapters using Trimmomatic (v.0.39), and quality control results were assessed using FastQC (v.0.12.1) (43). Clean reads were aligned to the reference genome. Qualified reads were aligned, sorted, and duplicates marked using BWA (v.0.7.17) and Picard (v.3.1.1) against the sheep reference genome (44, 45). SNP calling was performed using the Genome Analysis Toolkit GATK (v.4.2.5.0) with default parameters, and variant effects were annotated using SnpEff (v.5.2) (46). VCF files for the *KRT1* gene region were extracted using bcftools (v.1.19) and filtered using vcftools (v.0.1.16) with parameters –maf 0.05 and –max-missing 0.8 (47). Horn length-associated SNP loci within the *KRT1* gene region were extracted. Violin plots were generated to analyze the association between genotype mutations at these significant loci and the horn length trait in sheep.

## 3 Results

### 3.1 Expression of KRT1 gene in sheep with divergent horn phenotypes

**Figure 1:**
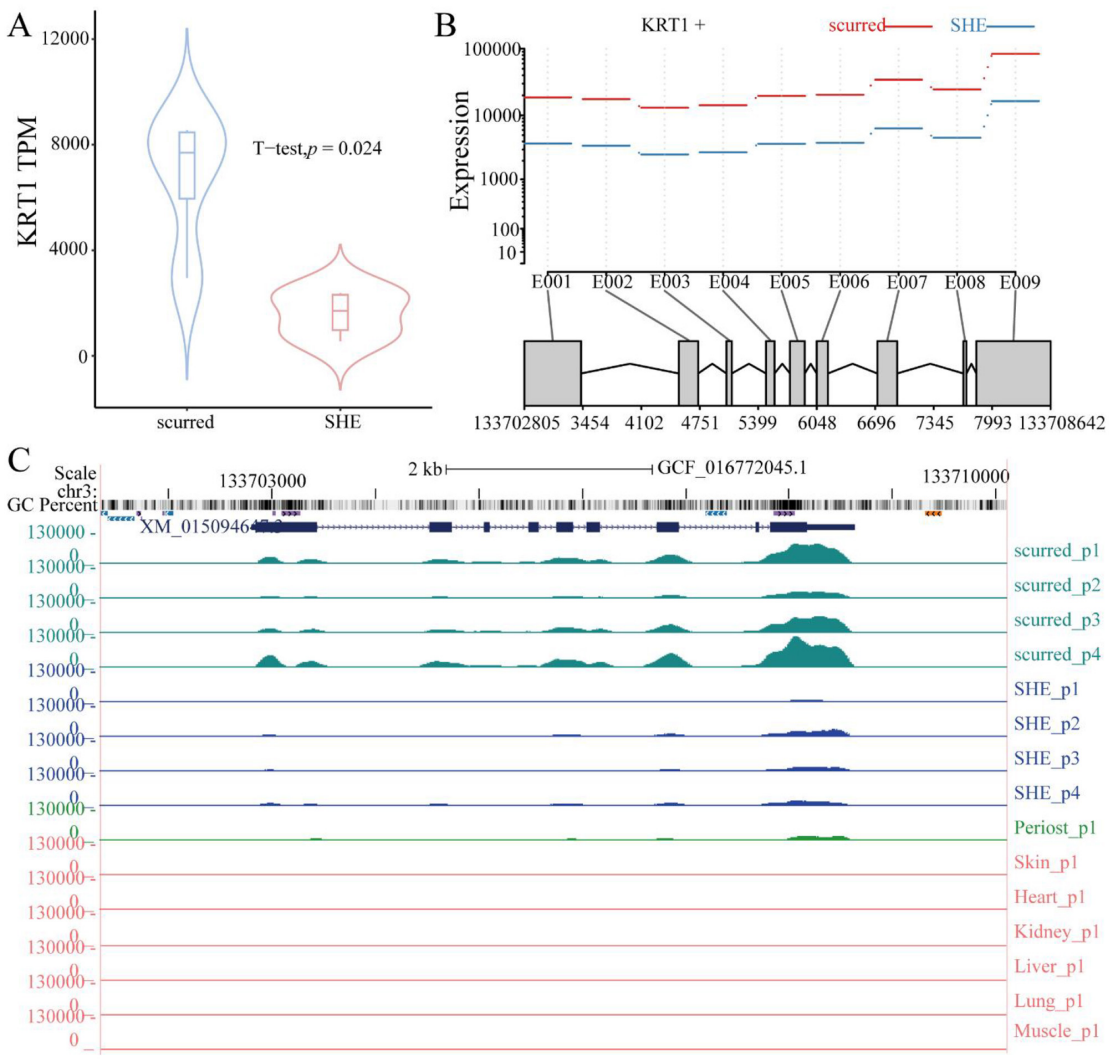
**(A)** Violin plot depicting differential *KRT1* gene expression between scurred (small-horned, *n* = 4) and SHE (large-horned, *n* = 4) horn tissues (*p* = 0.024, *T*-test). **(B)** Exon-specific expression of *KRT1* gene. *Y*-axis: Fitted expression estimates from GLM regression; *X*-axis: Exons E001–E009. Red and blue lines denote scurred and SHE groups, respectively. **(C)** UCSC Genome Browser view showing *KRT1* gene expression profiles, GC content, and repetitive elements in horn tissues (scurred/SHE) and seven somatic tissues. Tissue labels are indicated on the left.

Differential expression analysis revealed significantly higher *KRT1* gene transcript levels in the scurred group (small-horned phenotype) compared to the SHE group (large-horned phenotype; *p* = 0.024; Figure 1A). Exon-level resolution demonstrated elevated expression of all nine exons in scurred horn tissue, with the highest expression observed in exon 9 (E009; Figure 1B). Transcriptomic profiling via the UCSC Genome Browser (Figure 1C) delineated the expression patterns, GC content distribution, and repetitive element landscape of *KRT1* gene in horn tissues. Consistent with exon quantification data, all exons exhibited stronger expression signals in scurred vs. SHE samples, with maximal intensity at E009. Tissue-specific analysis across seven ovine tissues revealed pronounced *KRT1* gene expression in horn tissue, moderate levels in periosteum, lower expression in skin, and negligible detection in non-keratinized visceral organs (heart, liver, lung, kidney) and muscle. This expression hierarchy—horn tissue > periosteum > skin > non-keratinized viscera/muscle—highlights the structural congruence between horn keratinization and dermal-derived tissues. The concordant expression patterns in horn and periosteum support the hypothesis that horn morphogenesis involves skin-like keratinization processes governing both the keratinous sheath and bony core development.

### 3.2 The tissue-specific function of the KRT1 gene in sheep

**Figure 2:**
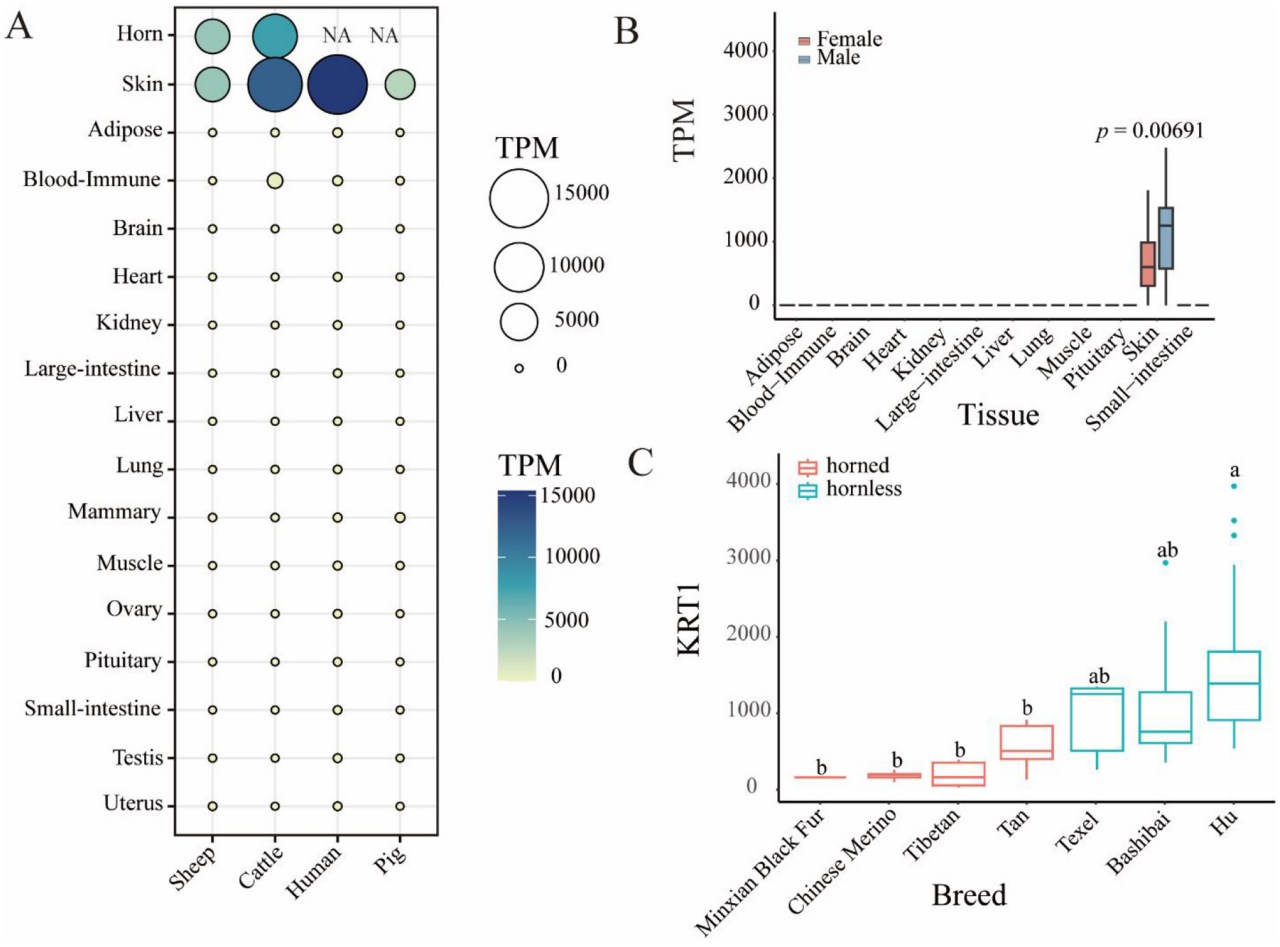
**(A)** The difference of RNA-seq expression of *KRT1* gene in different species and tissues. **(B)** The difference of RNA-seq expression of *KRT1* gene in tissues of Female and Male (*p* = 0.00691). **(C)** The difference of RNA-seq expression of *KRT1* gene in skin tissues of horned and hornless sheep.

To gain deeper insights into the cross-species expression profile of the *KRT1* gene, we examined its expression in sheep, pigs, humans, and cattle. Results (Figure 2A) revealed that *KRT1* gene is highly expressed in the skin tissue of all four species, with the highest expression level observed in human skin compared to bovine and ovine skin. Notably, significant *KRT1* gene expression was also detected in both sheep horn and cattle horn tissues, with higher expression levels in cattle horn than in sheep horn. Utilizing publicly available RNA-seq data, we further analyzed the sex-specific expression differences of *KRT1* gene across various sheep tissues (Figure 2B). The analysis demonstrated that *KRT1* gene expression is markedly tissue-specific, being detectable predominantly in skin tissue. Crucially, within skin tissue, *KRT1* gene expression levels were significantly higher in male individuals compared to females (*p* = 0.00691). Furthermore, to assess the consistency of *KRT1* gene expression across diverse sheep breeds, we compared its expression levels between horned and hornless sheep breed groups (Figure 2C). Among the hornless breeds, Hu Sheep exhibited the highest *KRT1* gene expression. Conversely, within the horned breeds, Minxian Black Fur Sheep showed the lowest expression. Overall, the expression level of *KRT1* gene was relatively higher in the hornless breed group compared to the horned breed group, with the difference being statistically significant, aligning with its potential biological role in horn development.

### 3.3 KRT1 protein evolutionary analysis

**Figure 3:**
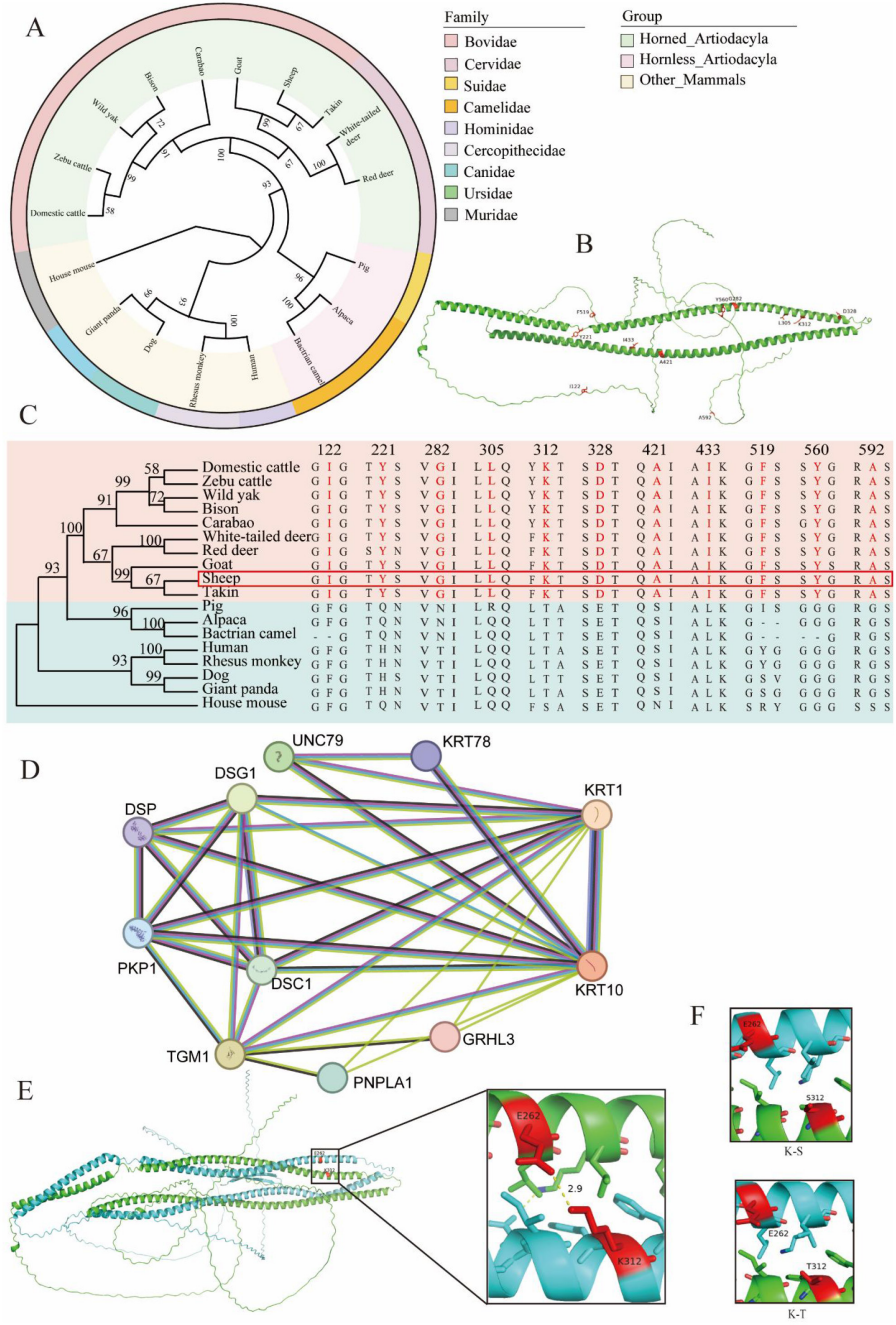
**(A)** Circular phylogenetic tree of KRT1 proteins from 18 species, with inner ring colored by group and outer ring colored by family. **(B)** 3D structure of the KRT1 protein highlighting the specific amino acid loci. **(C)** Comparative diagram of homologous KRT1 amino acid sequences from 18 species, with loci specific to horned artiodactyls marked in red. **(D)** Protein-protein interaction network of KRT1 in sheep. **(E)** Schematic diagram of the interaction between loci E262 of the sheep KRT1 protein and loci K312 of the KRT10 protein. **(F)** Schematic diagram illustrating the disruption of the hydrogen bond between KRT1 and KRT10 due to amino acid changes at loci 312 of the KRT1 protein.

A phylogenetic tree of KRT1 protein sequences from sheep and 17 other species was constructed using the NG method in MEGA11 software (Figure 3A). The results indicate that the KRT1 protein sequences are most closely related between sheep and other horned artiodactyls, suggesting structural similarity of the KRT1 protein among horned animals. Concurrently, the KRT1 amino acid sequences from these 18 species were compared, and 11 divergent amino acid loci specific to horned artiodactyls were identified (Figure 3C). Amino acid loci 122, 221, 282, 305, 312, 328, 421, 433, 519, 560, and 592 are identical in horned artiodactyls but differ in other mammals. These results demonstrate the structural similarity of the KRT1 protein among horned animals. These conserved loci may play crucial roles in horn growth and development. Furthermore, it was found that amino acid loci 282, 305, 312, 328, 421, and 433 are located on the α-helix of the central rod domain of the KRT1 protein, while the other loci reside on linkers, with loci 221, 519, and 560 being aromatic rings (Figure 3B).

**Figure 4:**
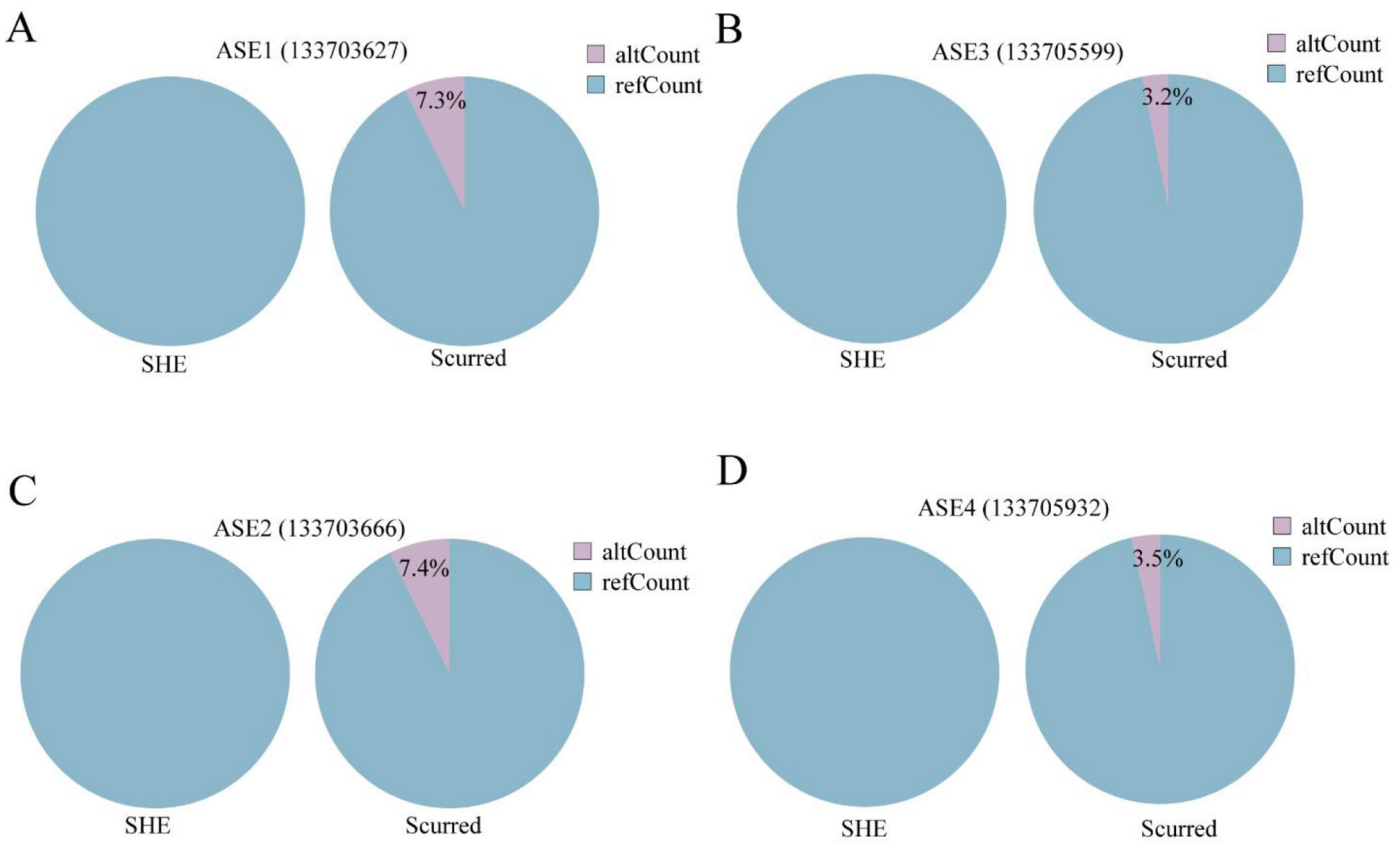
**(A)** Pie chart of differential ref/alt allele counts at ASE1 (chr3:133703627) between Scurred and SHE groups; blue = ref allele counts, purple =alt allele counts. **(B)** Pie chart of differential ref/alt allele counts at ASE1 (chr3:133705599) between Scurred and SHE groups; blue = ref allele counts, purple =alt allele counts. **(C)** Pie chart of differential ref/alt allele counts at ASE1 (chr3:133703666) between Scurred and SHE groups; blue = ref allele counts, purple =alt allele counts. **(D)** Pie chart of differential ref/alt allele counts at ASE1 (chr3:133705932) between Scurred and SHE groups; blue = ref allele counts, purple = alt allele counts.

The STRING database was used to predict the protein-protein interaction (PPI) network of KRT1 with other proteins in sheep (Figure 3D). Interactions were observed between KRT1 and proteins KRT10, KRT78, UNC79, GRHL3, DSC1, DSG1, PNPLA1, TGM1, PKP1, and DSP. Among these, KRT10 showed the strongest connection to KRT1. The study also identified a specific divergent amino acid sequence region involved in the interaction between KRT1 and KRT10 proteins. Specifically, the K312 amino acid loci of the KRT1 protein interacts with the E262 loci of the KRT10 protein via a hydrogen bond with a strength of 2.9 Å (Figure 3E). Based on the divergent amino acid loci specific to horned artiodactyls (referenced from Figure 4B), position 312 in hornless animals is occupied by T or S. It was observed that substitution of K312 with either T or S disrupts the hydrogen bond between K312 and E262 (Figure 3F). This further underscores the importance of the KRT1-KRT10 interaction in horned artiodactyls. This specific amino acid loci may play a vital role in horn growth and development.

### 3.4 Allele-specific expression (ASE) of KRT1 gene

Four critical allele-specific expression (ASE) loci were identified from 30 ASE (Supplementary Table S4) loci of the *KRT1* gene: ASE1 (chr3:133703627), ASE2 (chr3:133703666), ASE3 (chr3:133705599), and ASE4 (chr3:133705932), all located within introns. Analysis of ref/alt allele counts at these four ASE loci in SHE and Scurred groups (Figure 4) revealed a consistent pattern: ref allele counts markedly exceeded alt allele counts in the Scurred group; conversely, alt alleles were completely absent (zero counts) in the SHE group, where only ref alleles were expressed. This systematic bias toward ref allele expression—particularly the complete absence of alt alleles in the horned phenotype—indicates strong negative selection against alt alleles at these loci during horn development. The coordinated ASE pattern across these four intronic regulatory elements suggests their collective role in regulating *KRT1* expression and horn type determination.

### 3.5 Screening for potential functional variants in the KRT1 gene

**Figure 5:**
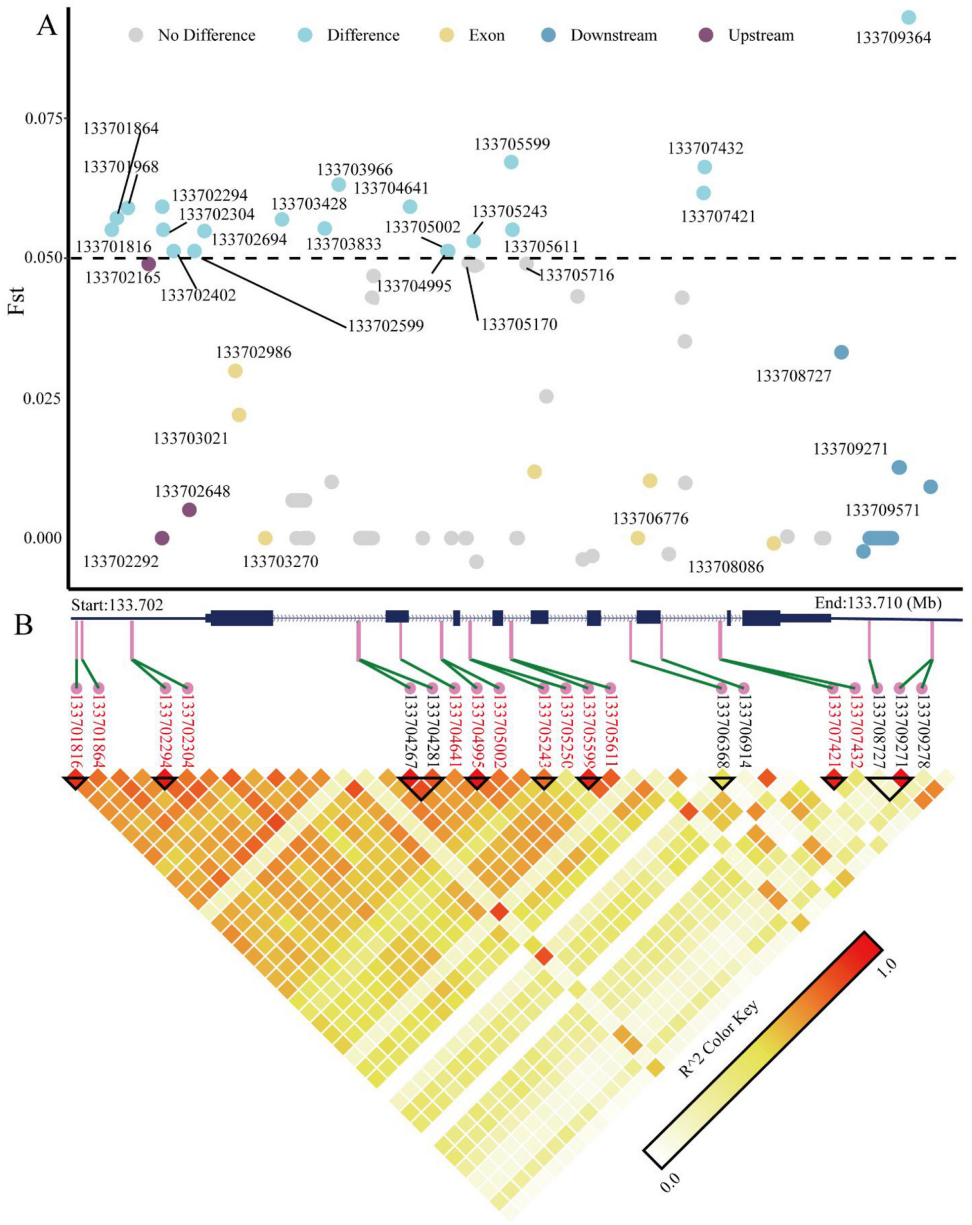
**(A)** Scatter plot showing the distribution of Fst values for all variant loci across the *KRT1* gene region. Loci with Fst > 0.05 (Difference) are marked in blue; loci with Fst < 0.05 (no Difference) are marked in gray. The upstream region (Upstream) is marked in purple; the downstream region (Downstream) is marked in dark blue; exonic regions (Exon) are marked in yellow. **(B)** Linkage disequilibrium (LD) plot for SNP loci within the *KRT1* gene. Darker block colors represent higher degrees of LD (measured as *r*^2^).

We analyzed the population differentiation index (Fst) values of variant loci within the *KRT1* gene across different sheep breeds (Figure 5A) and identified 20 loci exhibiting high selection signals (Fst > 0.05). This indicates significant population differentiation at these loci between large-horned and small-horned breeds, suggesting their potential association with horn formation or phenotypic variation. Through functional annotation of SNPs across the 199 variant loci in the *KRT1* gene region, we prioritized 32 potential functional SNPs (Table 1): SNPs 1–8 are located in the upstream promoter region. SNP 32 is located in the downstream region of the gene. SNPs 13–14, 16–21, 23, 26–27 are located within intronic regions. These potential functional SNPs generally display higher Fst values, further supporting their significant differentiation between large-horned and small-horned populations. This suggests they may play key roles in the transcriptional regulatory network or gene expression regulation governing horn development. Within the exonic regions of the *KRT1* gene, a total of 11 SNPs were identified (SNPs 9–12, 15, 22, 24–25, 28–31): six of these are synonymous mutation loci (SNPs 22, 24, 25, 28). These loci exhibit low Fst values, indicating minimal differentiation across populations, likely due to neutral selection. Notably, SNPs 12 and 15, despite being synonymous mutations, show relatively high Fst values. They might be involved in regulation by influencing codon usage bias or mRNA stability. Three SNP loci harbor missense mutations: SNP9 (p. Gly44Ala), SNP10 (p. Gly56Ser), SNP31 (p. Gly139Ser). Although the Fst values for these missense variants do not indicate significant differentiation, they may still exert functional effects on protein structure or function within specific populations, holding potential biological significance. Additionally, SNPs 29 to 31 are located within the 3′ untranslated region 3′ UTR. While their Fst values are not high and their population frequencies are low, their functional roles warrant further validation in larger sample cohorts.

**Table 1:** Potential SNP functional loci of *KRT1* gene.

**Info**	**Position**	**Mutation type**	**FST**	**Ref**	**Alt**
SNP1	133701816	upstream_gene_variant	0.05508	A	G
SNP2	133701864	upstream_gene_variant	0.05716	G	A
SNP3	133701968	upstream_gene_variant	0.05897	A	C
SNP4	133702294	upstream_gene_variant	0.05919	A	G
SNP5	133702304	upstream_gene_variant	0.05508	A	C
SNP6	133702402	upstream_gene_variant	0.05127	G	T
SNP7	133702599	upstream_gene_variant	0.05127	G	A
SNP8	133702694	upstream_gene_variant	0.05485	G	A
SNP9	133702986	missense_variant	0.02987	G	C
SNP10	133703021	missense_variant	0.02201	G	A
SNP11	133703270	missense_variant	0.00000	G	A
SNP12	133703428	synonymous_variant	0.05692	T	C
SNP13	133703833	intron_variant	0.05531	T	C
SNP14	133703966	intron_variant	0.06315	C	T
SNP15	133704641	synonymous_variant	0.05919	T	C
SNP16	133704995	intron_variant	0.05127	C	T
SNP17	133705002	intron_variant	0.05127	T	C
SNP18	133705243	intron_variant	0.05306	C	T
SNP19	133705250	intron_variant	0.04867	A	G
SNP20	133705599	intron_variant	0.06717	A	G
SNP21	133705611	intron_variant	0.05508	A	G
SNP22	133705820	synonymous_variant	0.01186	T	C
SNP23	133705932	intron_variant	0.02532	C	T
SNP24	133706776	synonymous_variant	0.00021	T	C
SNP25	133706914	synonymous_variant	0.01026	A	G
SNP26	133707421	intron_variant	0.06165	A	T
SNP27	133707432	intron_variant	0.06628	G	A
SNP28	133708086	synonymous_variant	0.00094	A	C
SNP29	133708214	3_prime_UTR_variant	0.00028	C	T
SNP30	133708537	3_prime_UTR_variant	0.00000	C	T
SNP31	133708562	3_prime_UTR_variant	0.00000	T	G
SNP32	133709364	downstream_gene_variant	0.09358	T	C

To further explore the genetic architecture of the *KRT1* gene and potential selection signals, we performed linkage disequilibrium (LD) analysis on these SNPs, identifying nine haplotype blocks. These blocks collectively encompass 13 SNPs with Fst > 0.05, specifically: SNP1 (chr3:133701816), SNP2 (chr3:133701864), SNP4 (chr3:133702294), SNP5 (chr3:133702304), SNP15 (chr3:133704641), SNP16 (chr3:133704995), SNP17 (chr3:133705002), SNP18 (chr3:133705243), SNP19 (chr3:133705250), SNP20 (chr3:133705599), SNP21 (chr3:133705611), SNP26 (chr3:133707421), SNP27 (chr3:133707432). In the LD plot (Figure 5B), the blocks representing the pairwise LD relationships among these SNPs show significantly darker coloration, indicating strong LD. These regions likely constitute genetic units closely associated with horn phenotype traits.

### 3.6 Association analysis between horn length and genotypes

**Figure 6:**
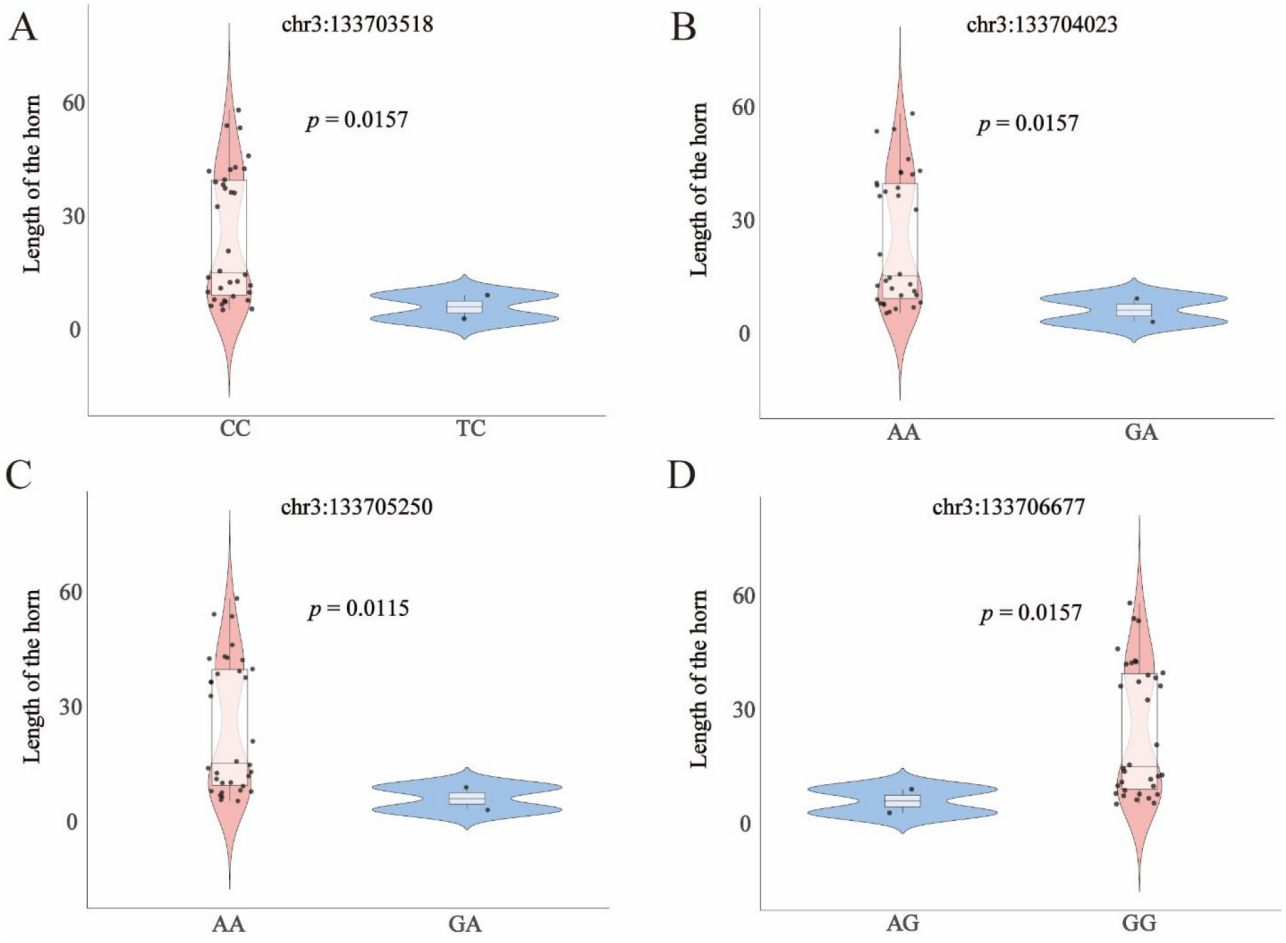
**(A)** Violin plot of horn length genotypes at chr3:1337303518, with *p*-value calculated by *T*-test (*p* = 0.0157). **(B)** Violin plot of horn length genotypes at chr3:1337304023, with *p*-value calculated by *T*-test (*p* = 0.0157). **(C)** Violin plot of horn length genotypes at chr3:1337305250, with *p*-value calculated by *T*-test (*p* = 0.0115). **(D)** Violin plot of horn length genotypes at chr3:1337306677, with *p*-value calculated by *T*-test (*p* = 0.0157).

To investigate loci significantly associated with horn length within the *KRT1* gene region, we performed association analysis using the average length of the left and right horns from 35 Small-tailed Han sheep individuals as the horn length phenotype. This analysis identified four SNPs significantly associated with horn length: chr3:133703518, chr3:133704023, chr3:133705250, and chr3:133706677 (Supplementary Table S5). At each of these four loci, individuals carrying the wild-type homozygous genotype exhibited significantly greater mean horn length compared to those carrying the heterozygous genotype (*p* < 0.05) (Figure 6): At chr3:133703518, the wild-type homozygous CC genotype had significantly longer horn than the heterozygous TC genotype (*p* = 0.0157). At chr3:133704023, the wild-type homozygous AA genotype had significantly longer horn than the heterozygous GA genotype (*p* = 0.0157). At chr3:133705250, the wild-type homozygous AA genotype had significantly longer horn than the heterozygous GA genotype (*p* = 0.0115). At chr3:133706677, the wild-type homozygous GG genotype had significantly longer horn than the heterozygous AG genotype (*p* = 0.0157). These results indicate that the wild-type homozygous genotype is associated with increased horn length, while the heterozygous genotype is associated with decreased horn length at these four loci. This suggests that the wild-type homozygous genotype may be the genotype associated with promoting horn growth. Notably, no individuals carrying the mutant homozygous genotype were detected for any of these four loci in our sampled population. We speculate that under certain conditions, these missing mutant homozygous genotypes may be lethal.

## 4 Discussion

Keratin 1 (KRT1) plays a pivotal role in maintaining the structural integrity of the skin. In the upper epidermis, KRT1 forms heterodimers with KRT10, assembling into intermediate filaments that provide mechanical strength (48). Significantly, homozygous nonsense mutations in KRT1 (c.457C>T, p. Gln153; c.33C>G, p. Tyr11) have been linked to epidermolytic palmoplantar keratoderma (EPPK) with knuckle pads, resulting from nonsense-mediated mRNA decay and loss of KRT1 protein (13). Furthermore, intergenic variation near *KRT1* correlates with the migratory capacity of human epidermal keratinocytes (HEKs) during wound healing (49), underscoring the critical function of *KRT1* in skin development and homeostasis. Given that horns are skin-derived structures, with growth originating from the dermis and subcutaneous layers as demonstrated by Corner bud tissue transplantation studies in young calf and caprids (50), and considering the skin-specific high expression of *KRT1* gene observed across species, we hypothesized that *KRT1* may regulate horn development by influencing the growth and differentiation of keratinocytes within the skin.

Our RNA-seq analysis revealed significantly elevated *KRT1* gene expression in the scurred group compared to the SHE group in sheep (*p* = 0.0024). We propose that this *KRT1* gene overexpression in scurred individuals contributes to localized thickening of the keratinous horn sheath, potentially inhibiting the longitudinal growth of the underlying horn core. As structural components forming the cytoskeleton of keratinocytes, keratins directly impact epithelial differentiation and cornification–key processes in horn sheath formation (51). This aligns with prior research in humans and model organisms, where *KRT1* gene mutations disrupt keratin filament assembly, leading to hyperkeratotic disorders like palmoplantar keratoderma (52), characterized by abnormal epidermal thickening analogous to the keratinization process in horn development. Our findings extend this mechanistic link to ovine horn morphogenesis, suggesting that *KRT1* gene overexpression in scurred group accelerates keratinocyte differentiation and matrix hardening, thereby restricting horn size. The consistency of this model among different sheep breeds (the expression of *KRT1* gene in hornless related breeds is higher than that in horned breeds) further suggests its evolutionary conservation in the regulation of horn development. Additionally, we observed significantly higher *KRT1* gene expression in ram skin compared to ewe skin (*p* = 0.00691), consistent with the observation that males typically possess larger, more robust horn structures requiring reinforced frontal bones to accommodate growth and withstand increased mechanical stress during physical interactions (53).

Phylogenetic analysis of KRT1 protein sequences across sheep and 17 other species identified several horned-animal-specific amino acid residues (e.g., K312) located within the central α-helical rod domain. This domain is essential for intermediate filament formation (54), and mutations within the H1 and α-helical rod domains of K1/K10 are known to cause bullous congenital ichthyosiform erythroderma (55, 56), highlighting the functional importance of this region in keratinization. Other identified specific residues, often aromatic amino acids, reside in low-complexity, aromatic-rich segments (LARKS) within the head or tail domains. LARKS facilitate molecular interactions crucial for keratin intermediate filament assembly (57), suggesting these loci may represent potential regulatory points for horn development. Protein-protein interaction network analysis (STRING database) confirmed KRT10 as the primary interactor with KRT1. KRT1 (basic, Type II) pairs with KRT10 (acidic, Type I) to form heterodimers (48), which self-assemble into antiparallel, staggered tetramers, ultimately forming intermediate filaments through longitudinal and lateral interactions (48, 58). The KRT1-KRT10 heterodimer interface, stabilized in part by the Cys401K10 disulfide bond, forms a half-staggered antiparallel tetrameric complex (57). Notably, our analysis suggests that the horned-animal-specific residue K312 in KRT1 may form a key hydrogen bond with E262 in KRT10. Mutations at K312 to S/T are predicted to disrupt this bond, indicating that the conservation of K312 is likely critical for the stability of the KRT1/KRT10 complex and, consequently, for proper keratin structure formation.

Utilizing public data, this study identified 20 mutation loci with FST > 0.05 within the *KRT1* gene and its flanking 1,000 bp regions, from which 32 potential functional loci were selected. These functional loci, prioritized either due to high FST values or specific selection criteria, are hypothesized to influence horn morphology and size by modulating gene expression or altering protein function. Subsequent linkage disequilibrium (LD) analysis delineated nine haplotype blocks encompassing 13 SNPs with FST > 0.05, suggesting these regions may harbor genetic units critically associated with horn type traits. Association analysis using self-measured average horn length data from Small Tail Han sheep revealed four loci (chr3:133705250, chr3:133704023, chr3:133706677, chr3:133703518) showing significant associations with horn length phenotypic variation. At these loci, the wild-type homozygous genotype was significantly associated with longer horns, while the heterozygous genotype corresponded to shorter horn lengths. This finding parallels observations by Zhao et al. (59). In their study on Gansu Alpine Fine-wool sheep, two SNPs (SNP1: C.-7G/C, SNP2: C.1500G/A) within the KRT71 gene, associated with wool traits, also showed that individuals homozygous for the wild-type allele had a significantly longer mean staple length (MSL) than heterozygous individuals (*p* < 0.05). Notably, our study work failed to detect individuals homozygous for the mutant allele at these significantly associated loci, leading to the hypothesis that this genotype may confer lethality under certain conditions. Furthermore, this study identified four loci exhibiting allele-specific expression (ASE). At these ASE loci, the reference allele (ref) count was significantly higher in the SHE group compared to the scurred group, while the alternative allele (alt) count was zero in the SHE group. This pattern strongly suggests that these loci may cooperatively regulate horn size development. Hereditary ichthyoses, known to be caused by dominant-negative mutations in keratin genes such as *KRT1, KRT2*, and *KRT10*, disrupt epidermal keratinization (60). Drawing an analogy to this established pathological mechanism, we speculate that the specific allelic variations identified in the *KRT1* gene in this study (i.e., the FST > 0.05 loci, association loci, and ASE loci) may similarly interfere with epidermal keratinization or related processes, thereby influencing horn morphogenesis and size regulation.

This study provides valuable insights for sheep breeding programs targeting horn phenotypes. Future research should integrate single-cell RNA sequencing (scRNA-seq), epigenomic analyses, and histological examinations for multi-omics profiling. This approach will help identify specific cell types and locate critical regulatory elements of *KRT1* gene influencing horn development. Functional validation through CRISPR-Cas9-mediated mutagenesis or *in vitro* assays will be essential to confirm the transcriptional regulatory effects of prioritized SNPs and elucidate the precise molecular mechanisms by which *KRT1* gene governs horn morphogenesis in sheep.

## 5 Conclusions

This study demonstrates a significant association between the *KRT1* gene and horn size in sheep. Expression analysis revealed a relatively higher level of KRT1 expression in the scurred sheep group. Cross-species investigations further confirmed the high tissue specificity of *KRT1*, with robust expression observed predominantly in the skin and horn tissue of multiple species, including sheep, cattle, pigs, and humans. In-depth analysis of the amino acid sequence encoded by the *KRT1* gene and its protein structure identified 11 amino acid loci exhibiting specificity in horned animals. Crucially, an interaction was discovered between lysine at position 312 (K312) of the KRT1 protein and glutamate at position 262 (E262) of the KRT10 protein. This specific interaction loci may play a pivotal role in keratin filament network formation and horn development. Furthermore, this study integrated RNA-seq and whole-genome sequencing (WGS) datasets. Through subsequent analysis of SNP variations in the *KRT1* gene, allele-specific expression (ASE), and population differentiation index (Fst), a series of potential functional regulatory loci and genetic variants significantly associated with horn length were successfully identified. These loci likely influence horn size and morphology (horned, polled, or scurred) by modulating gene function or expression levels. Based on these findings, we propose that these loci serve as potential molecular markers for horn type traits (including horn length and presence/absence) in sheep. In conclusion, this research provides novel insights into the biological function of the *KRT1* gene in ovine horn development and establishes a crucial theoretical foundation for future molecular breeding strategies targeting horn phenotype in sheep.

## Data Availability

The original data presented in the study are publicly available. The Tibetan sheep RNA-seq data can be found in the NCBI repository under accession number PRJNA1003277. Publicly available datasets analyzed in this study are cited within the article and their sources are provided in the Methods section.
